# Improvement in clinical outcome and quality of life after arthroscopic ankle arthrodesis in paralytic foot drop

**DOI:** 10.1186/s13018-023-03691-y

**Published:** 2023-03-14

**Authors:** Fahmy Samir Fahmy, Mohammad Abdalla Abd El Salam, Hossam Fathi Mahmoud

**Affiliations:** Department of Orthopedic Surgery, Faculty of Medicine, Zagazig University, Sharkia, Egypt

**Keywords:** Foot-drop, Paralytic, Arthroscopic arthrodesis, Ankle

## Abstract

**Background:**

Paralytic foot-drop is a disabling deformity that results from nerve or direct muscle injuries. Palliative surgeries such as tendon transfer and ankle arthrodesis are reserved for permanent deformity, with the arthroscopic technique had not been widely studied before. This study aims to evaluate the clinical outcome and quality of life after arthroscopic ankle fusion of paralytic foot-drop deformity.

**Materials and Methods:**

The patients who were retrospectively enrolled in this study underwent arthroscopic ankle fusion for paralytic foot-drop deformity between March 2017 and December 2021. The American Orthopedic Foot and Ankle Society (AOFAS) ankle–hindfoot score and Cumberland Ankle Instability Tool (CAIT) were the measures used for clinical assessment. To judge the union, serial plain radiographs of the ankle were obtained. The preoperative and postoperative means were analyzed utilizing a two-tailed paired t-test, with a p value of less than 0.05 indicating statistical significance.

**Results:**

This study included 21 consecutive patients with a mean follow-up of 35.09 ± 4.5 months and a mean age of 41.5 ± 6.1 years. Highly significant improvements were observed between the preoperative and final follow-up means of the AOFAS score (from 57.6 ± 4.6 to 88.3 ± 2.7) and CAIT (from 12.1 ± 2.2 to 28.9 ± 1.01; p ˂ 0.00001 for both). All patients attained radiographic union and resumed their previous occupations without reporting serious adverse effects.

**Conclusions:**

Arthroscopic ankle fusion is an effective, minimally invasive palliative surgery for patients suffering from permanent paralytic foot-drop deformity. This technique was shown to provide good functional and radiologic outcomes without significant complications.

**Level of evidence:**

Retrospective cohort; level of evidence (IV).

**Supplementary Information:**

The online version contains supplementary material available at 10.1186/s13018-023-03691-y.

## Introduction

Foot-drop deformity is described as a lack of active ankle dorsiflexion resulting in major functional impairment for patients. Possible causes of this condition include systemic diseases, neurologic injuries, and local muscle injuries. Sciatic and common peroneal nerve palsies are the most common traumatic mononeuropathies that produce such deformities [1–4].

Patients with foot-drop deformity complain of motor weakness (loss of active ankle dorsiflexion and eversion), impaired sensation on the dorsum of the foot, and abnormally high steppage gait. In addition, the loss of ankle stability during walking, with a high risk of repeated falls, impairs patients’ daily activities and diminishes their quality of life [5, 6].

Conservative measures, direct nerve repair, and nerve grafting are valuable treatment modalities for early nerve injuries. Although there have been remarkable improvements in surgical skills and equipment used in peripheral nerve surgeries in the past decades, direct repair, or grafting results in a failure to regain motor function in 64% of sciatic and 45%–55% of common peroneal nerve lesions [7, 8].

Long-standing foot-drop deformity (> 12 months) is an indication for palliative surgery. Posterior tibial tendon transfer through the interosseous membrane to the dorsal foot aims to improve motor function with the restoration of active dorsiflexion. However, ankle fusion is indicated in patients with ankle arthritis, poor skin condition, weak tibialis posterior muscle, or failure of previous tendon transfer surgery [9].

The goal of ankle fusion is to produce a painless, stable ankle during walking. Various ankle fusion procedures have been mentioned in the literature [10]. Open ankle arthrodesis is the most common technique used among orthopaedic surgeons. Wound-healing problems, infection, and nonunion are the major complications associated with open surgery [11].

With the development of optics and instruments, arthroscopic ankle fusion has gained popularity since it was first described in 1983 [12]. Most published studies recommended the arthroscopic technique in patients with minimal ankle deformities (less than 15° in the coronal plane) and compromised skin conditions [13]. Fewer wound-healing complications, shorter hospital stays, faster rehabilitation, and shorter union time are the main advantages of arthroscopic ankle fusion over open techniques [14, 15].

To our acquaintance, no previously published research has studied the short-term clinical outcomes and quality of life after arthroscopic ankle arthrodesis of paralytic foot-drop deformity. Our study aims to assess the functional results after arthroscopic ankle fusion of permanent foot-drop, including patient satisfaction. Our hypothesis is that arthroscopic ankle arthrodesis would provide a significant improvement in the functional outcome, enabling a return to previous activities.

## Patients and methods

This retrospective study involved 21 patients who suffered from permanent paralytic foot drop. Patients were enrolled after obtaining approval from our institutional review board (IRB) and informed consent from all participants. The study was conducted between March 2017 and December 2021 at Zagazig University hospital. Our series enrolled 16 men and 5 women, with a mean age of 41.5 ± 6.1 years. All patients underwent a period of conservative treatment that entailed physical therapy and the use of ankle–foot splints for a minimum of 1 year before surgical intervention. After that, they were treated with arthroscopic ankle fusion. The criteria for eligibility were as follows: (1) sciatic nerve lesions confirmed by electromyography (EMG) and nerve conduction velocity (NCV) lasting more than 12 months; (2) lumbar disc injuries with persistent foot-drop; and (3) previous traumatic open muscle injuries of the leg associated with poor skin condition. We excluded patients with early nerve lesions, Charcot ankle, foot-drop deformity with good tibialis posterior muscle power (grade 4 or 5), fixed ankle deformities, previous ankle fractures, ankle arthritis (Kellgren-Lawrence grades 3 and (4), subtalar arthritis, skeletal immaturity, and deficient medical files. Table 1 summarizes the characteristics of the included patients.

**Table 1:** The patients' demographic criteria

Age (years)	
Mean ± standard deviation (SD)	41.5 ± 6.1
Range (years)	(29–52)
Sex	
Male	16 (76.2%)
Female	5 (23.8%)
Side of injury	
Right	13 (61.9%)
Left	8 (38.1%)
Occupation	
Employer	8 (38.1%)
Factory worker	4 (19.04%)
Homemaker	3 (14.3%)
Plumber	1 (4.8%)
Farmworker	1 (4.8%)
Electrician	1 (4.8%)
Mechanics	1 (4.8%)
Builder	1 (4.8%)
Carpenter	1 (4.8%)

Data are presented as number (n), percentage (%), and mean ± SD (range)

### Surgical technique

**Fig. 1:**
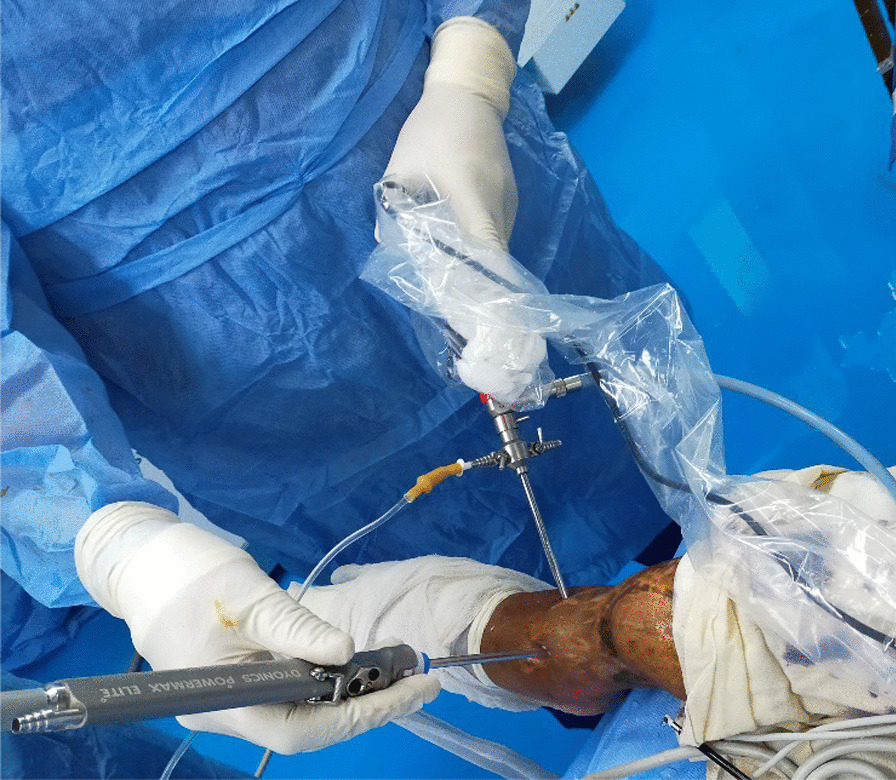
Patient position and portals for arthroscopic ankle fusion

Preoperatively, 1 g of cefuroxime intravenous was administered 30 min before the induction of anesthesia. All procedures were performed by one surgeon under spinal or epidural anesthesia with a well-padded thigh tourniquet inflated to 300–350 mmHg. The patient was lying supine on the operating table with the foot hanging over the edge. (Fig. 1).

**Fig. 2:**
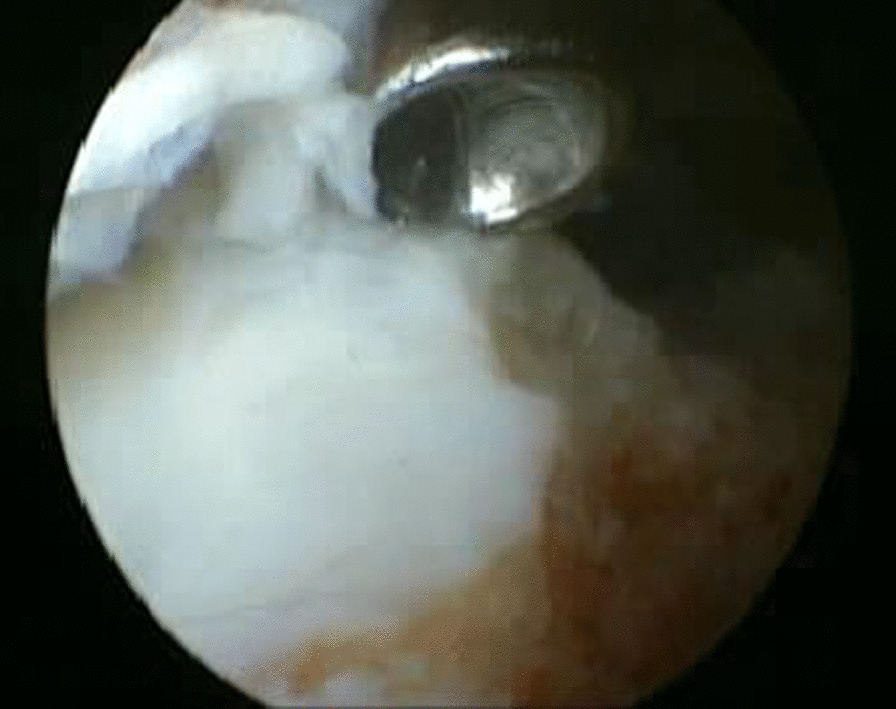
Arthroscopic view showing the removal of articular cartilage from superior surface of the talar dome with a small curette

**Fig. 3:**
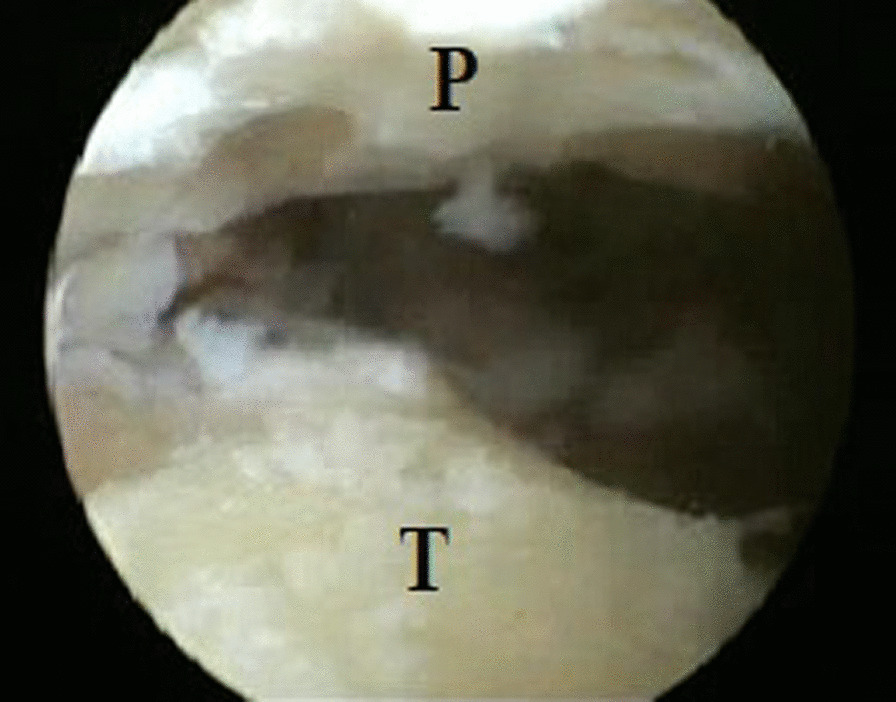
Arthroscopic view after complete removal of articular cartilage from the distal tibia and body of the talus. P; plafond, and T; talar dome

**Fig. 4:**
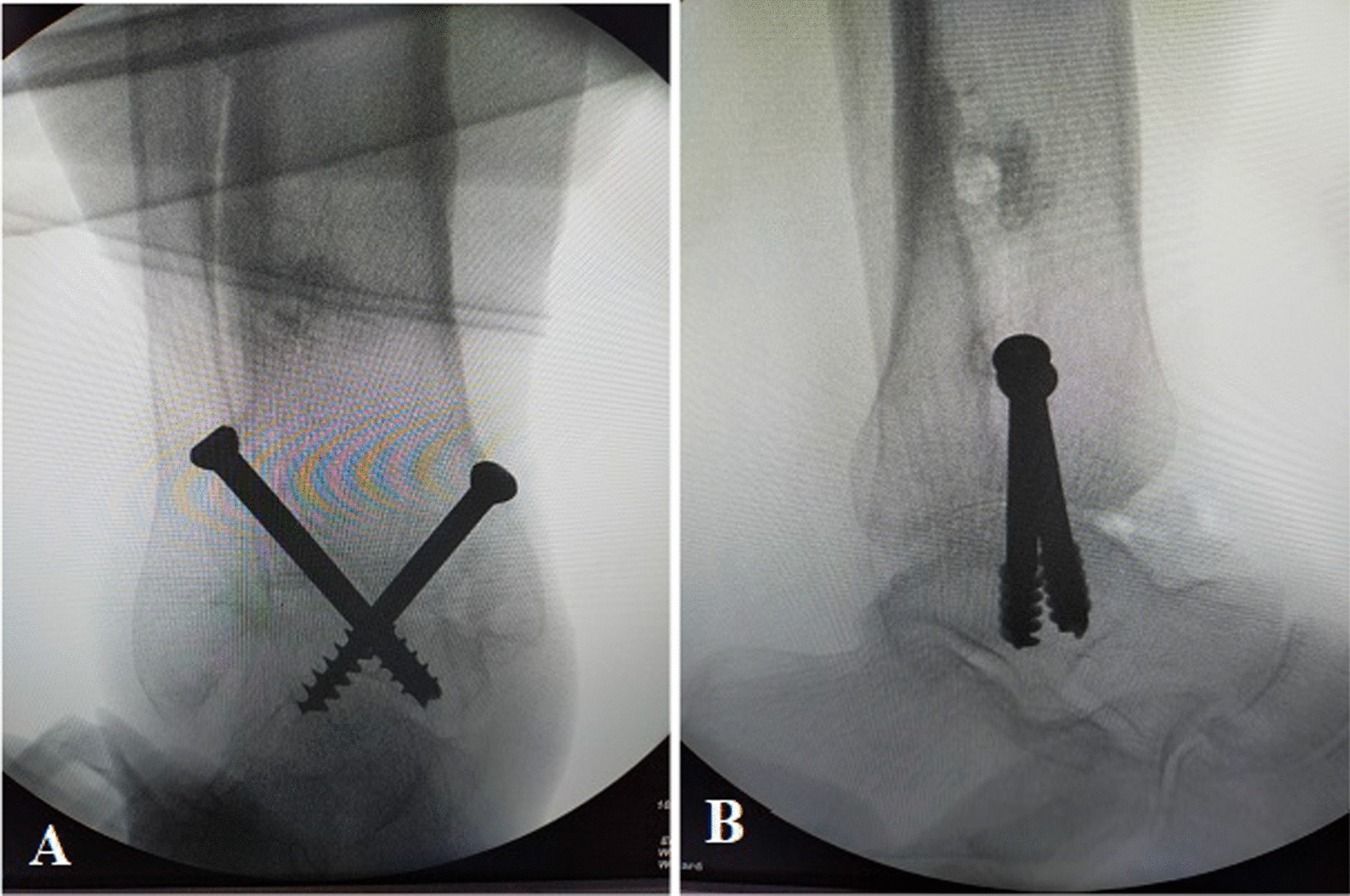
Intraoperative fluoroscopic imaging to check the final position of the construct fixing and compressing the arthrodesis site. **a** Anteroposterior, and **b** Lateral views

The joint was instilled with 20 mL of saline to distend the joint capsule through the soft spot medial to the tibialis anterior tendon. The arthroscope was introduced into the ankle joint through the anteromedial portal, and the anterolateral portal was established under arthroscopic vision. The hyaline cartilage covering the tibial plafond and the dome of the talus were ablated with the aid of small curettes and a motorized shaver blade (Stryker, USA) with a radius of 3.5 mm (Fig. 2). In addition, the articular cartilage of the medial malleolus was removed, and the lateral gutter was debrided only to facilitate compression of the joint. A small burr with a 3.5-mm diameter cleared the remaining cartilage down to the subchondral region, making small microfracture pits within the surfaces of the bone. After completion of articular cartilage debridement (Fig. 3), two percutaneous 6.5-mm cannulated crossing screws were used for fixation under the guidance of a C-arm image intensifier. They were applied from the medial and lateral sides of the distal tibia and advanced down to the body of the talus, avoiding penetration of the subtalar joint with the foot aligned in 90° dorsiflexion, neutral varus–valgus, and neutral rotation (Fig. 4). The final position of the foot and screws was checked, and the wounds were stitched with an application of plaster of Paris splint below the knee.

### Postoperative care and follow-up

Patients continued antibiotics for 2 days after surgery, with the removal of the stitches after 2 weeks. The ankle was held in a below-knee cast without weight bearing for 6 weeks. Partial weight bearing in a walker splint was permitted after the radiographic appearance of bridging trabeculae. Full weight bearing was allowed after 3 months, and the patients were instructed to wear half-boot shoes with a rocker sole during occupation to provide support for the subtalar and midtarsal joints. Plain radiographs of the ankle were obtained at 0, 8, 12, 16, and 20 weeks postoperatively to assess the progress of union and then at the last follow-up to check for the position of the screws and exclude subtalar arthritis. The American Orthopedic Foot and Ankle Society (AOFAS) ankle–hindfoot score [16] and Cumberland Ankle Instability Tool (CAIT) [17] were recorded for functional assessment at the last follow-up. The quality of life and patient satisfaction were assessed by the physical and mental health level in the Patient-Reported Outcomes Measurement Information System (PROMIS Global-10) [18], with a maximum score of 20 points for each.

### Statistical analysis

At first, the Kolmogorov–Smirnov test was employed to verify the data normality. The numerical values were presented as means and standard deviations, with a 95 percent confidence interval calculated for the means. We estimated the difference between the preoperative and final postoperative means of the AOFAS, CAIT, and PROMIS using a paired t-test. P ˂ 0.05 indicated statistical significance for all tests. The post hoc analysis demonstrated that the statistical power was 93.6% for an alpha error of 0.05 and a large effect size difference for two dependent means using the G-Power system version 3.1. For statistical analysis, we utilized the Statistical Package for Social Sciences (IBM, version 16.0, Inc., Chicago, IL).

## Results

Following the elimination of one case with incomplete records and one case that was lost during follow-up, our study retrospectively analyzed the outcome of arthroscopic ankle fusion in 21 patients with persistent paralytic foot-drop deformity with an average age of 41.5 ± 6.1 years. The mean follow-up period was 35.09 ± 4.5 (range, 25–42 months). No serious complications were reported in this study. Only one patient experienced a transient superficial peroneal nerve sensory deficit, which recovered within 12 weeks after surgery. No cases of nonunion, implant failure, or subtalar arthritis were reported at the last follow-up.

The reported causes of permanent foot-drop deformity in our study were as follows: 12 patients with old sciatic nerve injuries, 4 with previous lumbar disc lesions, and 5 with previous open muscle injuries with poor skin condition. The mean duration from the initial injury to surgery was 15.7 ± 2.5 months (range, 12–21 months).

The mean operative time was 57.3 ± 8.3 min, with an average postoperative hospital stay of 2.1 days (range, 1–4 days).

**Fig. 5:**
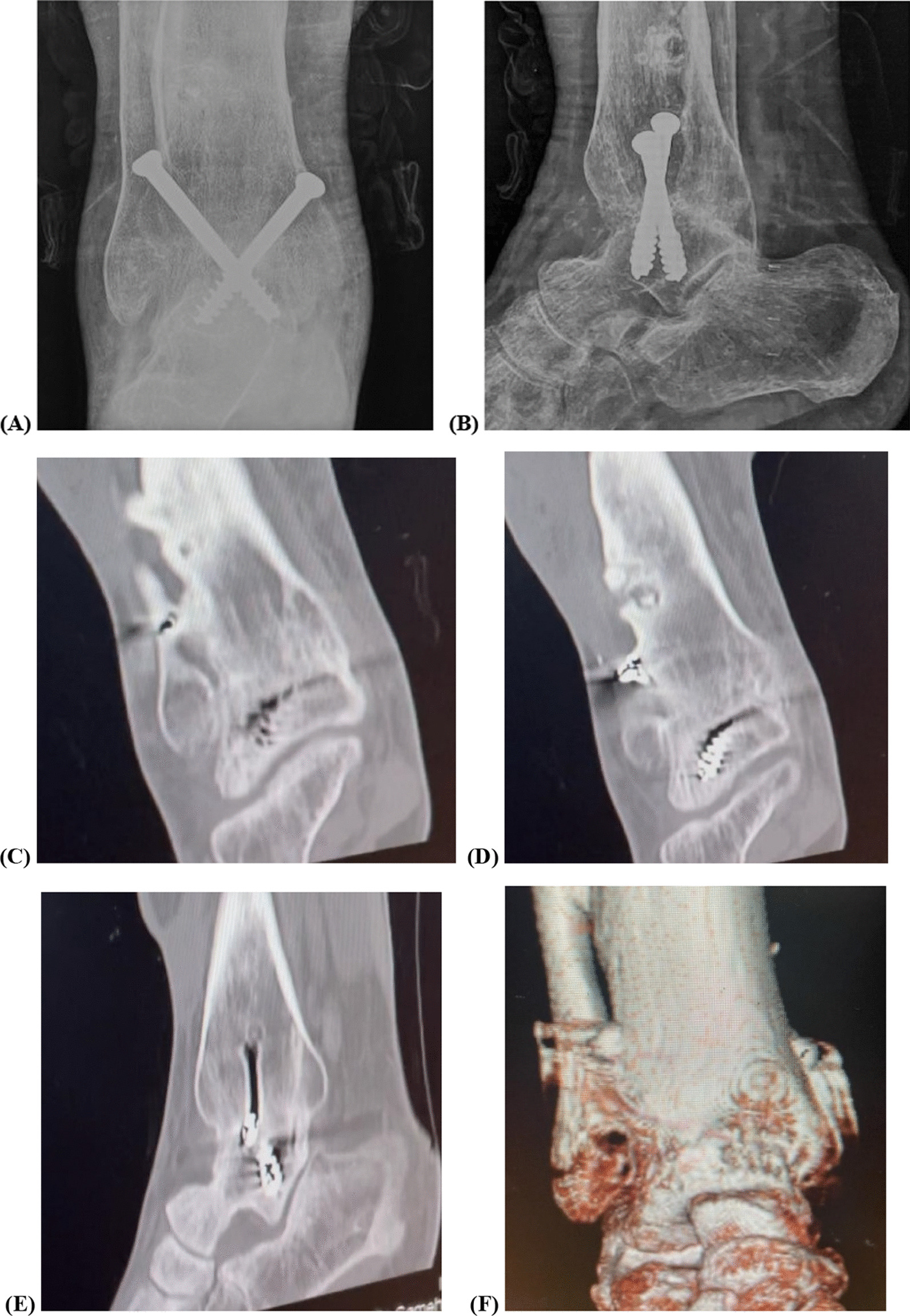
Plain radiographs show radiographic union at 5 months postoperatively in the anteroposterior (**a**) and lateral views (**b**), and the CT images demonstrate full consolidation at 9 months (**c**–**f**)

The preoperative mean of AOFAS ankle–hindfoot score was improved at the last follow-up with a statistically significant difference (from 57.6 ± 4.6 to 88.3 ± 2.7; P < 0.00001). A statistically significant improvement was observed in CAIT from 12.1 ± 2.2 preoperatively to 28.9 ± 1.01 at the final postoperative follow-up (P < 0.00001) (Table 2). All patients achieved radiographic union with an average time of 12.8 weeks (range, 8–20 weeks) (Fig. 5).

**Table 2:** Preoperative and final postoperative clinical scores

	Preoperative mean	Preoperative 95% CI	Final postoperative mean	Final postoperative 95% CI	P-value
AOFAS ankle score	57.6 ± 4.6	55.5–59.7	88.3 ± 2.7	87.1–89.5	< 0.00001
CAIT for ankle stability	12.1 ± 2.2	11.1–13.1	28.9 ± 1.01	28.4–29.3	< 0.00001
PROMIS					
Physical health score	10.7 ± 1.5	10.01–11.3	17.4 ± 1.6	16.6–18.1	< 0.00001
Mental health score	10.7 ± 1.64	9.9–11.4	17.8 ± 1.2	17.2–18.3	< 0.00001

*CI* confidence interval, *AOFAS* American Orthopaedic Foot and Ankle Society, *CAIT* Cumberland Ankle Instability Tool. *PROMIS* Patient-Reported Outcomes Measurement Information System. *P* ˂0.01 indicates highly significant results

The level of physical status in PROMIS prior to surgery was obviously increased from 10.7 ± 1.5 points to 17.4 ± 1.6 points at the final follow-up. Also, the mental health was obviously improved from 10.7 ± 1.64 points preoperatively to 17.8 ± 1.2 points at the last follow-up. At the last follow up, the patients were satisfied with their quality of life (Additional file 1) and returned to the preinjury activity level without functional deficit, with a mean time to return to occupation of 16 ± 2.3 weeks.

## Discussion

Our study demonstrated a significant improvement in the final postoperative clinical scores. All patients were pleased with the outcomes, as they regained ankle stability, resumed their pre-injury activity level, and exhibited a good quality of life.

Most published studies have shown poor compliance and unsatisfactory functional outcomes of conservative management, especially in young, active patients [7]. Tibiotalar fusion and muscle transfer are salvage procedures for persistent paralytic foot-drop deformity after failure of conservative measures or early nerve surgeries [9]. Cho et al. [19] stated that 17 patients with paralytic foot-drop had only 33% of normal ankle extension strength restored after tibialis posterior tendon transfer. Ankle fusion is indicated in patients suffering from peripheral nerve or direct muscle injuries who are not candidates for tendon transfer. The main contraindications of dynamic muscle transfer are weak tibialis posterior tendon, advanced ankle arthritis, and unfavorable local soft-tissue condition [9].

Arthroscopic tibiotalar arthrodesis is a minimally invasive procedure that provides a fusion rate comparable to that of open techniques, with fewer wound-healing problems, lower infection rates, reduced postoperative pain, and earlier rehabilitation [20, 21]. The success of arthroscopic fusion is dependent on the complete removal of the articular cartilage, adequate mechanical strength of the fixation method, and proper ankle positioning [22].

This research was the first to investigate the clinical outcome of arthroscopic tibiotalar fusion in paralytic foot-drop; thus, we considered the few available trials of posttraumatic arthroscopic ankle fusion for comparison.

For the fixation, we used two partially threaded, cannulated 6.5-mm screws. There is no consensus among the published studies regarding the number of screws necessary for rigid fixation [23]. Danawi et al. [24] and Winson et al. [25] used two cancellous 6.5-mm screws for fixation, achieving a solid union of 91% and 92% in their series, respectively. Clifford et al. [26] concluded that there was no difference in the mechanical strength between anterior, or lateral plating, and compression screw constructs.

We reported a mean operative time of 57.3 ± 8.3 min, which was shorter than the 99 ± 16.4 min recorded by Townshend et al. [27], 104 ± 35 min reported by Gougoulias et al. [28], and 140.5 ± 22.2 min reported by Wang et al. [29]. The possible explanation for our result was that operating on a normal ankle with preserved joint space was much easier and less time-consuming than fusion in posttraumatic arthritic ankles. In addition, our average operative time was lower than what Woo et al. [30] reported in 56 patients who had open fusion (107.9 ± 31.2 min).

The mean postoperative hospital stay in this study was 2.1 days (range, 1–4 days), which was consistent with the mean recorded by Townshend et al. [27] (2.5 days) and Woo et al. [30] (2.1 days). Winson et al. [25] and Gougoulias et al. [28] reported longer mean hospital stays of 4 days (range, 1–21 days) and 3.7 days (1–27 days), respectively, owing to the larger number of patients and more recorded postoperative complications in their series. A longer hospital stay was reported by Peterson et al. [31] in ten cases of open ankle arthrodesis, with a mean stay of 4.5 ± 2.45 days.

We noticed a statistically significant increase in the postoperative AOFAS ankle-hind foot score with a final mean of 88.3 (range, 83–93), which was comparable with the results of Xiaojun et al. [21] (86 ± 5) and better than what was recorded by Wang et al. [29] (77.7 ± 3.8) and Woo et al. [30] (78.9 ± 18.9). In addition, we noted superior results compared with open surgeries by Napiontek and Jaszczak [33] (73.5; range, 60–100), Wang et al. [29] (75.8 ± 4.5), and Woo et al. [30] (68.9 ± 24.7). In a recent meta-analysis conducted by Bai et al. [33], the authors stated that arthroscopic ankle fusion was advantageous to the open technique in terms of mean hospital stay, intraoperative blood loss, and AOFAS ankle score.

The bony union is judged as clinically painless and a stable ankle on weight bearing with radiologic evidence of bridging bony trabeculae without signs of implant failure [34]. Several studies have stated that the arthroscopic ankle technique has a better fusion rate in a shorter time as compared with open surgeries due to the reduced periosteal stripping with preservation of the local vascularity around the ankle [28, 33]. In our study, the mean time of union was close to that reported by Ferkel et al. [12] (an average of 11.8 weeks) and Wang et al. [29] (12.4 ± 1.9 weeks). In the study by Wang et al. [29] of 26 patients who underwent open ankle arthrodesis, the authors reported a longer mean time of fusion (14.6 ± 3.4 weeks). Our study recorded a 100% union rate, which was comparable with that of Peterson et al. [31] (100%) and Meng et al. [35] (100%), and our results were better than those achieved in open surgeries by Quayle et al. [36] (79.3%).

All patients in our series returned to work within a mean time of 16 ± 2.3 weeks, which was better than that reported by De Vriese [20] (20 ± 2 weeks). Trouillier et al. [37] reported that footwear modification following ankle fusion diminished the stresses on the subtalar and midtarsal joints, with improvement of the gait pattern. Therefore, the patients in this study were advised to modify their footwear during work.

Our patients were satisfied with the ultimate results without reporting postoperative subtalar arthritis due to the short-term follow-up. However, Fuchs et al. [38] recorded 13 cases of subtalar arthritis in 18 patients who underwent ankle fusion and were followed up for 23 years; their patients remained satisfied and recommended the surgery for young patients.

We recorded one minor complication of temporary sensory neuropraxia of the superficial peroneal nerve, which resolved within 12 weeks after the operation, with an overall complication rate of 4.7%. Our results were consistent with the rate reported by Li et al. [39] (5.5%) and were better than the complication rates among patients who underwent open surgeries recorded by several authors in a meta-analysis by Bai et al. [33].

This study was not without limitations. First, the number of eligible patients was insufficient for generalizability of the results; a larger sample size is required to increase statistical power and the reliability of the findings. Second, there was no control group for comparison with other treatment options. In addition, the follow-up period was not enough to identify the remote complications, such as subtalar and midtarsal arthritis. Finally, because it was a retrospective analysis with no data on the radiographic alignment angles, a prospective study is needed to obtain these measures.

## Conclusion

Arthroscopic ankle fusion of paralytic foot-drop deformity provided a successful clinical outcome with a statistically substantial improvement in the functional AOFAS score, PROMIS, and CAIT at a minimum of 2-year follow-up. It resulted in a rigid, stable ankle, enabling the patients to resume their daily activities with minor complications. The patients achieved full radiographic union with a high level of satisfaction at the end of follow-up. Further studies will be warranted to evaluate the functional results of the arthroscopic fusion in other neuromuscular disorders.

## Supplementary Information

**Additional file 1.** A male patient experienced paralytic foot drop, compromised soft tissue, and knee stiffness due to a previous united, open floating knee fracture. He was dependent on two crutches and retired. After surgery, he can walk unaided with an improved gait pattern and return to work.

## Data Availability

The required datasets of the current study are available from the corresponding author on reasonable request.

## Abbreviations

AOFAS: American Orthopaedic Foot and Ankle Society
CAIT: Cumberland ankle instability tool
PROMIS: Patient-reported outcomes measurement information system
SD: Standard deviation
CI: Confidence interval
g: Gram
mmHg: Millimeter mercury
h: Hour
mL: Milliliter
mm: Millimeter
min: Minute
