# Supplementary material for: Improvement in clinical outcome and quality of life after arthroscopic ankle arthrodesis in paralytic foot drop
Source: J Orthop Surg Res. 2023 Mar 14;18:202. doi: 10.1186/s13018-023-03691-y (PMC10015676; doi:10.1186/s13018-023-03691-y)
Supplement: Supplementary file 1 — Additional file 1. A male patient experienced paralytic foot drop, compromised soft tissue, and knee stiffness due to a previous united, open floating knee fracture. He was dependent on two crutches and retired. After surgery, he can walk unaided with an improved gait pattern and return to work. [file 13018_2023_3691_MOESM1_ESM.pptx]

## Slide 1
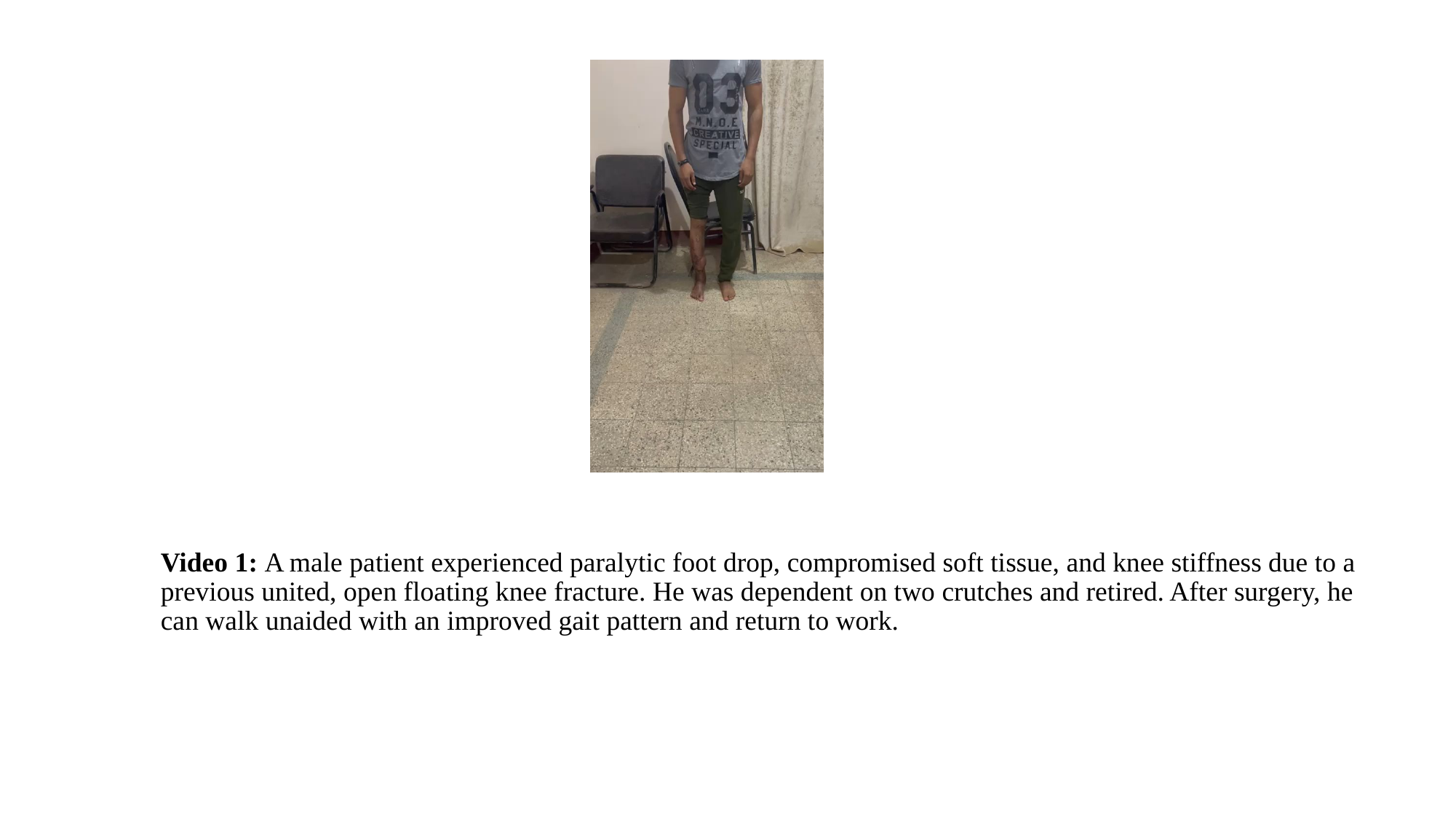

# Video 1: A male patient experienced paralytic foot drop, compromised soft tissue, and knee stiffness due to a previous united, open floating knee fracture. He was dependent on two crutches and retired. After surgery, he can walk unaided with an improved gait pattern and return to work.
